# Fears and Perception of the Impact of COVID-19 on Patients With Lung Cancer: A Mono-Institutional Survey

**DOI:** 10.3389/fonc.2020.584612

**Published:** 2020-10-14

**Authors:** Chiara Catania, Gianluca Spitaleri, Ester Del Signore, Ilaria Attili, Davide Radice, Valeria Stati, Letizia Gianoncelli, Stefania Morganti, Filippo de Marinis

**Affiliations:** ^1^Division of Thoracic Oncology, IEO, European Institute of Oncology IRCCS, Milan, Italy; ^2^Division of Epidemiology and Biostatistics, IEO, European Institute of Oncology IRCCS, Milan, Italy; ^3^Division of Early Drug Development for Innovative Therapies, IEO European Institute of Oncology IRCCS, Milan, Italy; ^4^Department of Oncology and Hemato-Oncology, University of Milan, Milan, Italy

**Keywords:** lung cancer, COVID-19, coronavirus disease 2019, survey, fears, quality of life, patient’s perception

## Abstract

In February 2020, Italy became one of the first countries to be plagued by the SARS-CoV-2 pandemic, COVID-19. In March 2020, the Italian government decreed a lockdown for the whole country, which overturned communication systems, hospital organization, and access to patients and their relatives and carers. This issue had a particular regard for cancer patients. Our Thoracic Oncology Division therefore reorganized patient access in order to reduce the risk of contagion and, at the same time, encourage the continuation of treatment. Our staff contacted all patients to inform them of any changes in treatment planning, check that they were taking safety measures, and ascertain their feelings and whether they had any COVID-19 symptoms. To better understand patients’ fears and expectations of during the pandemic period, we created a nine-question interview, administered from April to May 2020 to 156 patients with lung cancer. Patients were classified by age, sex, comorbidity, disease stage, prior treatment, and treatment type. The survey showed that during the pandemic period some patients experienced fear of COVID-19, in particular: women (55% vs. 33%), patients with comorbidities (24% vs. 9%), and patients who had already received prior insult (radiotherapy or surgery) on the lung (30% vs. 11%). In addition, the patients who received oral treatment at home or for whom intravenous treatment was delayed, experienced a sense of relief (90% and 72% respectively). However, only 21% of the patients were more afraid of COVID-19 than of their cancer, in particular patients with long-term (> 12 months) vs. short-term cancer diagnosis (28% vs. 12.5%, respectively). Furthermore, the quarantine period or even just the lockdown period alone, worsened the quality of life of some patients (40%), especially those in oral treatment (47%). Our data demonstrate how lung cancer patients are more afraid of their disease than of a world pandemic. Also this interview indirectly highlights the clinician’s major guiding principle in correctly and appropriately managing not just the patient’s expectations of their illness and its treatment, but also and especially of the patient’s fears.

## Introduction

In December 2019, a novel coronavirus, known as Severe Acute Respiratory Syndrome Coronavirus 2 (SARS-CoV-2), was identified in Wuhan (China) as the cause of coronavirus disease 2019 (COVID-19) (1, 2). The SARS-CoV-2 infection spread worldwide, and the World Health Organization (WHO) declared COVID-19 a pandemic on March 11, 2020 (3). At the time of writing, a total of 13,287,651 cases of COVID-19 have been recorded worldwide, causing 577,954 deaths (a 4.3% mortality rate). Unfortunately, we are far from the contagion’s peak. In Italy, 243,149 cases of COVID-19 have been identified, with 34,984 deaths (mortality rate of 14.3%) (4). The symptoms of COVID-19 are commonly fever, anosmia, disgeusia, diarrhea, cough, fatigue, anorexia and myalgias (2). Some patients may develop acute respiratory distress syndrome, requiring intensive care and mechanical ventilation and a few may even die (5, 6). Factors favoring severe COVID-19-related complications are age over 65 years and the presence of chronic health conditions (e.g., cardiovascular disease, diabetes mellitus, and obesity). However, severe cases have occurred in individuals without known risk factors (6). Two prospective trials have investigated the impact of COVID-19 in cancer patients: CCC19 (all cancers) and TERAVOLT (only thoracic cancers) (7, 8). In the CCC19 cohort study, 121 (13%) deaths were recorded out of 928 of patients with cancer (82% solid tumors) and the risk factors for mortality were: older age, male sex, ever smoker, number of comorbidities, performance status of 2 or more, active cancer, and prior administration of azithromycin plus hydroxychloroquine (7). The TERAVOLT trial, presented at the ASCO 2020 meeting update, reported 141 deaths (35.5%) out of 400 thoracic cancer patients. About 80% of these were related to COVID-19. Factors associated with a high risk of death were: age, performance status, and the presence of chronic health conditions.

Moreover, prior administration of chemotherapy (alone or in combination with immunotherapy), steroids, and anticoagulant therapy may increase risk of death (8). Following the initial spread of COVID-19 in Italy, a lockdown was imposed on 23 February, 2020 by the Italian government, covering those regions of Italy mostly hit by the pandemic spread. On March 11, 2020 the lockdown was extended to the entire country. Milan became difficult to reach for those who lived far away due to the restrictions on who could enter and exit the city. Only essential activities e.g., cancer treatment, were allowed (with the compulsory quarantine of patients on returning to their region). All healthcare institutes rearranged their internal guidelines. We introduced proactive management and containment measures that helped us better identify those patients with suspected symptoms related to COVID-19, thereby limiting the virus spread throughout the hospital. These measures are reported elsewhere (9). National and international guidelines were published to help manage patients with cancer, especially those with lung cancer (10–12). To protect our lung cancer patients from the risk of COVID-19 infection, our Thoracic Oncology Division decided to delay treatments mainly for those who were particularly vulnerable and those who had to travel from afar. Measures included deferring follow-up visits and sending cancer medications to the patients’ homes. Our staff also contacted all patients to ascertain how they were feeling, whether they had experienced any symptoms of COVID-19, whether they used masks and observed all safety measures and to communicate to them any changes to appointments (9). We had varying responses to these calls; some accepted our recommendations and thanked us; others became irate for fear that the disease could progress. We therefore wanted to transform our perceptions derived from the outcome of these telephone conversations into something objective. We developed a qualitative survey to assess the following issues: their fear of falling ill with COVID-19 compared to the fear of their disease, how much the COVID-19 emergency had changed their lives, and whether they were more afraid of COVID-19 than of lung cancer. We wanted to understand the patients’ lived experiences regarding the pandemic situation and their disease. Since there were no available validated questionnaires that could answer our question, we decided to conduct guided interviews with patients during outpatient visits before their treatment. We asked the same questions to understand whether their main concern was: “my disease” or “coronavirus” and how they reacted when we contacted them about skipping a treatment. Here we present the results of these interviews with our patients.

## Materials and Methods

**Figure 1:**
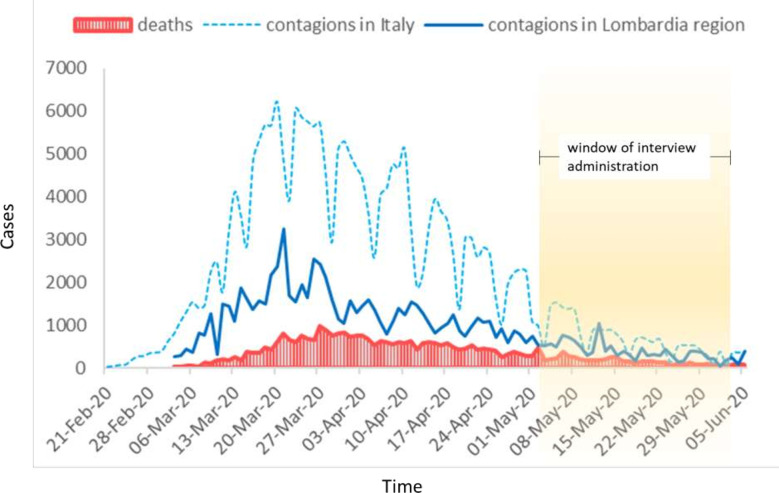
The graphic shows the evolution of contagions and deaths in Italy and in Lombardy during the pandemic period. The interval of all the interviews is represented in the graph.

We carried out this study from April 30, 2020 to May 29, 2020 (**Figure 1**). We included 156 patients with lung cancer who came to the European Institute of Oncology in Milan for cancer treatment, a medical examination, or a follow-up visit. These patients were asked nine questions during the interview carried out at the consultation that we usually conduct before our physical examination and prescribing cancer treatment. We decided to conduct a nine-question interview because there were no existing questionnaires that could evaluate the items that we wanted to investigate. The same physician (the First Author CC) conducted all the interviews on the day the patients came to our institute. The same doctor who conducted the interviews (the First Author CC) noted down the patients’ freely-given explanations of all their answers to the questions.

### Structured Interview

Our team of thoracic oncologists selected the questions based on continuous discussions we held during our team meetings. Before drawing up the nine questions for the interview, our team interrogated the US National Library of Medicine’s biomedical literature database PubMed, and reviewed all the pertinent literature. We did not find any validated questionnaires aimed at evaluating the real emotional impact of coronavirus in patients with lung cancer. Nine questions were drawn up, and all the interviews conducted by the doctors used the same questions. The possible replies to the first eight questions ranged from “*not at all*” to “*extremely*” or “*not evaluable*” (see below). The ninth question, “Are you more afraid of COVID-19 or your cancer?” is self-explanatory: “yes”, “no”, “both equally” (**Appendix 1**). We asked the same questions to understand whether their main concern was “their disease” or “coronavirus”, and how disturbing they found our communication about skipping a treatment. We developed a qualitative survey to assess: their fear of falling ill with COVID-19 compared to the fear of their disease; how much the COVID-19 emergency had changed their lives; whether they were more afraid of COVID-19 than lung cancer; and to understand whether our telephone calls to ascertain their state of health and communicate any postponements or cancellations of treatments and appointments had worried them or made them more secure and therefore happier.

### Participants

All the patients referred to the European Institute of Oncology in Milan for a visit or treatment for early/advanced/metastatic lung cancer from April 30, 2020, to May 29, 2020, were eligible. Patients were informed that an interview would be conducted, and we asked them if they would consent to answer some questions. All patients were free to accept or refuse the interview without it affecting the visit or treatment program. All patients were Italian and had a good understanding of the Italian language in order to be able to answer the questions.

### Administration of the Structured Interview

The same interview was given to all patients who came to the Day Hospital to receive intravenous treatment, all patients who came to the clinic to receive oral treatment (TKI: tyrosine kinase inhibitors) and all patients who came for check-ups or first visits and therefore had yet to start treatment. Those patients who did not receive a deferral of treatment or visits between February and March 2020 were not asked questions Q5 and Q6 in the interview. Lung cancer patients receiving oral therapy (TKI) in the period February and March 2020 skipped visits and outpatient check-ups. However, they still received care at home without visiting us by receiving phone calls. These patients were asked all of the questions set out in the interview. We felt that skipping the visit with their oncologist, not being able to discuss the side effects of the treatment, have a physical examination or see the CT images immediately, could be considered an alteration/delay compared to what they usually did in the pre-COVID- 19 period. We reasoned that this could have affected their fear and perception about the risk of contagion and their disease (less exposed to the risk of contagion but less monitored by specialists).

### Statistical Analysis

Patients’ characteristics and treatments were described by counts and percent, and age by mean and standard deviation (SD). Days from cancer diagnosis to structured interview administration were categorized into three levels (within three months, between three months and one year, and over one year). Answers to the structured interview questions from Q1 to Q9 were cross-tabulated against patient characteristics and treatments and tested for their association by the Fisher’s exact test, taking into account the missing data if their proportion was greater than 5% in any of the cells. A multivariable multinomial logistic regression analysis was performed for all questions, including only the significant factors at the univariate analysis. Answers were categorized into three levels: *not at all/a little* (reference level), *moderately* and *quite a bit/extremely*. The multivariable analysis results were presented as odds ratios (OR) and tabulated with 95% confidence intervals (95%CI). All tests were two-tailed and considered significant at the 5% level. All analyses were performed using SAS 9.4 (N.C., Cary USA).

## Results

During the study period, a total of 156 patients were interviewed. The clinical features of the included population are shown in **Table 1**. The median age was 68 years (range 23-91). Patients were predominantly male (55.8%). Most patients (69%) were from the Lombardy region of Italy. At the time of our interview, 138 (88%) of the patients were receiving systemic anticancer treatment, 83 patients (53%) intravenous treatment, and 55 patients (35%) were receiving oral tyrosine kinase inhibitors-TKIs) Moreover, most of the patients (87%) had advanced disease and less than 15% of patients were receiving curative treatment.

**Table 1:** Patient’s characteristics and Treatments, N = 156.

Characteristics		Statistics*^a^*
Age, years	Mean (SD)	66.5 (± 10.3)
	<75	129 (82.7)
	≥75	27 (17.3)
Months from cancer diagnosis	≤3	28 (18.0)
	(3–12]	32 (20.5)
	>12	96 (61.5)
Sex	Female	69 (44.2)
	Male	87 (55.8)
Histology	Adenocarcinoma	132 (84.6)
	Squamous carcinoma	10 (6.4)
	SCLC	9 (5.8)
	LCNEC	1 (0.6)
	Other	4 (2.6)
Current stage	Metastatic	136 (87.7)
	Locally advanced	14 (9.0)
	Early	6 (3.9)
ECOG PS	0	48 (30.8)
	1	108 (69.2)
Smoking status	Never	46 (29.5)
	Former*^b^*	75 (48.1)
	Current	35 (22.4)
Comorbidities	Cardio-pulmonary	114 (73.1)
Region	Lombardia	107 (69.0)
	Other regions	49 (31.0)
Previous surgery/pulmonary RT		73 (46.8)
Therapy setting	Metastatic	140 (89.7)
	Adjuvant/Neo-adjuvant	10 (7.7)
	CT/RT	6 (3.9)
Therapy	No therapy	18 (11.5)
	TKI	55 (35.3)
	Intravenous	83 (53.2)
	CT	30 (19.2)
	IO	35 (22.4)
	CT+IO	15 (9.6)
	CT+RT	3 (1.9)

a Statistics are: mean (± SD) for age, N (%) otherwise.

b Quit smoking for at least 12 months.

TKI, tyrosine kinase inhibitor; CT, chemotherapy; IO, immunotherapy; RT, radiotherapy; SCLC, small cell lung cancer; LCNEC, large cell neuroendocrine carcinoma; SD, standard deviation.

The structured interview is fully shown in **Appendix 1**.

**Table 2** shows the distribution of the answers in the overall interviewed population.

**Table 2:** Frequency distribution of answers to the structured interview, all patients N = 156.

Question	Level	N (column %)
Q1	Not at all/a little	86 (55.1)
	Moderately	33 (21.2)
	Quite a bit/extremely	31 (19.9)
	*missing*	6 (3.9)
Q2	Not at all/a little	94 (60.3)
	Moderately	35 (22.4)
	Quite a bit/extremely	22 (14.1)
	*missing*	5 (3.2)
Q3	Not at all/a little	70 (44.9)
	Moderately	45 (28.9)
	Quite a bit/extremely	32 (20.5)
	*missing*	9 (5.8)
Q4	Not at all/a little	92 (59.0)
	Moderately	37 (23.7)
	Quite a bit/extremely	19 (12.2)
	*missing*	8 (5.1)
Q5^a^	Not at all/a little	53 (82.8)
	Moderately	4 (6.3)
	Quite a bit/extremely	6 (9.4)
	*missing*	1 (1.6)
Q6^a^	Not at all/a little	20 (31.3)
	Moderately	8 (12.5)
	Quite a bit/extremely	34 (53.1)
	*missing*	2 (3.1)
Q7*^b^*	Not at all/a little	101 (73.2)
	Moderately	12 (8.7)
	Quite a bit/extremely	16 (11.6)
	*missing*	9 (6.5)
Q8	Not at all/a little	88 (56.4)
	Moderately	28 (18.0)
	Quite a bit/extremely	32 (20.5)
	*missing*	8 (5.1)
Q9	COVID	33 (21.2)
	Oncological disease	89 (57.1)
	Both equally	26 (16.7)
	*missing*	8 (5.1)

a Sample Size N = 64 (delayed patients only, see text for details).

b Sample Size N =138 (excluding subjects without therapy).

### Worries About COVID-19

#### Most Patients With Lung Cancer Report Not to Be Worried About COVID-19

In the first two questions (Q1 and Q2), patients were asked whether they were worried about COVID-19, at the beginning of the pandemic (Q1) and at the time of the interview (Q2). Most patients (55.1% in Q1 and 60.3% in Q2, respectively) reported *not at all/a little* worried about COVID-19, while only less than 20% reported being *quite a bit/extremely* worried (19.9% in Q1 and 14.1% in Q2, respectively) (**Table 2**). In univariate analyses, the patients’ gender, comorbidities, and previous thoracic local treatment, significantly affected the answer distribution to Q1 (p: 0.02, p: 0.006, p: 0.01, respectively). The observation of the answer distribution among the main three categories (*not at all/a little*, *moderately*, *quite a bit/extremely*), according to these patients’ characteristics, reveal a higher proportion of male compared to female patients (66.7% vs. 45.5%) reporting *not at all/a little* worried about COVID-19, whereas female patients were more likely to be *quite a bit/extremely* worried compared to males (28.8% vs. 14.3%). Of note, although the absence of worry remains the predominant answer for all patients, a higher proportion of patients with comorbidities or previous thoracic treatment, compared to those without, reported to be *quite a bit/extremely* worried about COVID-19 (23.7% vs. 9.5% and 30.1% vs. 10.8%, respectively) (**Table 3**; **Supplementary Tables 1****,** **2**). In the subgroup of patients receiving intravenous (IV) anticancer treatment (N= 83), the Q1 answer distribution was statistically different according to treatment type (chemotherapy containing regimens vs. immunotherapy alone, p: 0.02). In multivariate analyses, female sex and previous thoracic treatment confirmed to be associated in Q1 with higher probability of being *quite a bit/extremely* worried about COVID-19 compared to *not at all/a little* (OR 3.08, 95% CI 1.26–7.52, p: 0.01; OR 3.75, 95% CI 1.50–9.37, p< 0.001, respectively) (**Table 5**, **Supplementary Figure 1**).Similar findings were observed in Q2 with regard to gender and comorbidities affecting answer distribution in univariate analyses (p: 0.003, p: 0.01, respectively) (**Table 3**; **Supplementary Tables 1****,** **3**). In Q2, only female sex was associated with higher probability of being worried about COVID-19 (moderately: OR 3.43, 95% CI 1.51–7.79; *quite a bit/extremely*: OR 3.42, 95% CI 1.29–9.07) (**Supplementary Figure 1**, **Table 5**).

**Table 3:** Question number with a significant factor association to the answers to the structured interview.

Question	Factor	Level	N (row %)					p-value
			N	Not at all/A little	Moderately	Quite a bit/Extremely	*missing*	
Q1	Gender	Female	69	30 (45.5)	17 (25.8)	19 (28.8)	3 (4.4)	
		Male	87	56 (66.7)	16 (19.1)	12 (14.3)	3 (4.4)	**0.02**
	Comorbidity	No	42	20 (47.6)	14 (33.3)	4 (9.5)	4 (9.5)	
		Yes	114	66 (57.9)	19 (16.7)	27 (23.7)	2 (1.8)	**0.006**
	Previous surgery/pulmonary RT	No	83	52 (62.7)	17 (20.5)	9 (10.8)	5 (6.0)	
		Yes	73	34 (46.6)	16 (21.9)	22 (30.1)	1 (1.4)	**0.01**
	Therapy*^a^*	CT,CT+IO,CT+RT	48	30 (62.5)	12 (25.0)	6 (12.5)	0	
		IO	35	22 (62.9)	2 (5.7)	9 (25.7)	2 (5.7)	**0.02**
Q2	Gender	Female	69	31 (47.0)	22 (33.3)	13 (19.7)	3 (4.4)	
		Male	87	63 (74.1)	13 (15.3)	9 (10.6)	2 (2.3)	**0.003**
	Comorbidity	No	42	25 (59.5)	11 (26.2)	2 (4.8)	4 (9.5)	
		Yes	114	69 (60.5)	24 (21.1)	20 (17.5)	1 (0.9)	**0.01**
Q3	Therapy	No therapy	18	5 (29.4)	8 (47.1)	3 (17.7)	1 (5.9)	
		Intravenous*^b^*	83	48 (57.1)	22 (26.2)	10 (12.1)	3 (3.6)	
		TKI	55	17 (30.9)	15 (27.3)	18 (32.7)	5 (9.1)	**0.009*^c^***
	Months from cancer diagnosis	≤3	28	11 (39.3)	10 (35.7)	2 (7.1)	5 (17.9)	
		(3,12]	32	19 (59.4)	9 (28.1)	3 (9.4)	1 (3.1)	
		>12	96	40 (41.7)	26 (27.1)	27 (28.1)	3 (3.1)	**0.01**
Q4	Months from cancer diagnosis	≤3	28	13 (46.4)	8 (28.6)	2 (7.1)	5 (17.9)	
		(3,12]	32	24 (75.0)	5 (15.6)	2 (6.3)	1 (3.1)	
		>12	96	55 (57.3)	24 (25.0)	15 (15.6)	2 (2.1)	**0.03**
Q8	Therapy	Intravenous^c^	83	34 (70.8)	7 (14.6)	7 (14.6)	0	
		TKI	55	25 (45.5)	13 (23.6)	13 (23.6)	4 (7.3)	**0.04**

^a^N= 83 patients receiving intravenous treatment subgroup; ^b^Intravenous: CT,CT+IO,CT+RT,IO; ^c^Intravenous vs. TKI comparison (excluding No therapy level), p = 0.003;

CT, chemotherapy; IO, immunotherapy; RT, radiotherapy; TKI, tyrosine kinase inhibitors.

#### Patients With Lung Cancer Are More Worried by Their Oncologic Disease Than by COVID-19

Question 9 (Q9) specifically investigated which one of the diseases (COVID-19 vs. lung cancer) worried patients most. Eighty-nine patients (57%) reported being more worried by their lung cancer than by COVID-19, 33 patients (21%) were more worried by COVID-19 and 26 patients (17%) reported to be equally worried by the two conditions (**Table 2**).

In univariate analysis, factors significantly associated with answer distribution were: previous thoracic treatment (p = 0.003), systemic treatment type (intravenous vs. oral. P = 0.02) and time to cancer diagnosis (p = 0.006) (**Table 4**).

**Table 4:** Significant factor associations to the answers to the structured interview question 9.

Factor	Level	N (row %)					p-value
		N	COVID-19	Oncological Disease	Both equally	*missing*	
Previous Surgery/Pulmonary RT	No	83	11 (13.3)	48 (57.8)	16 (19.3)	8 (9.6)	
	Yes	73	22 (30.1)	41 (56.2)	10 (13.7)	0	**0.003**
Therapy	Intravenous*^a^*	83	5 (10.4)	30 (62.5)	11 (27.1)	2 (4.2)	
	TKI	55	14 (25.5)	35 (63.6)	4 (7.3)	2 (3.6)	**0.02**
Months from cancer diagnosis	≤3	28	2 (7.1)	20 (71.4)	5 (17.9)	1 (3.6)	
	(3,12]	32	4 (12.5)	14 (43.8)	10 (31.3)	4 (12.5)	
	>12	96	27 (28.1)	55 (57.3)	11 (11.5)	3 (3.1)	**0.006**

a Intravenous: CT, CT+IO, CT+RT, IO.

TKI, tyrosine kinase inhibitor; CT, chemotherapy; IO, immunotherapy; RT, radiotherapy.

In particular, despite the overall main worry about the oncological disease, 22 (30%) patients with previous thoracic treatment compared to 11 (13%) without, reported to be most worried by COVID-19, similarly 14 (25.5%) patients with Oral TKI treatment compared to 5 (10%) IV patients (p = 0.02) and 27 (28%) long-term lung (> 12 months) cancer diagnosis vs. 4 (12.5%) middle-term (3 to 12 months) vs. 2 (7%) short-term (≤ 3 months) (p = 0.006) lung cancer diagnosis reported to be most worried by COVID-19 (**Table 4**). At multivariable analysis, only patients with long-term vs. short-term cancer diagnosis were still significantly more worried by COVID-19 (p = 0.04), (**Supplementary Figure 1**, **Table 5**).

**Table 5:** Multivariable multinomial logistic regression analysis of distribution of answers to the structured interview.

Question		Answers compared to the reference level*^a^*	Odds Ratio (95% CI)	p-value
Q1	Female vs. Male	Moderately	1.73 (0.75–4.00)	0.20
		Quite a bit/Extremely	3.08 (1.26–7.52)	**0.01**
	Comorbidity vs. No comorbidity	Moderately	0.46 (0.19–1.10)	0.08
		Quite a bit/Extremely	2.85 (0.83–9.76)	0.10
	Previous surgery/pulmonary RT	Moderately	1.29 (0.56–2.96)	0.55
		Quite a bit/Extremely	3.75 (1.50–9.37)	**<0.001**
Q2	Female vs. Male	Moderately	3.43 (1.51–7.79)	0.003
		Quite a bit/Extremely	3.42 (1.29–9.07)	0.01
	Comorbidity vs. No comorbidity	Moderately	0.98 (0.40–2.39)	0.97
		Quite a bit/Extremely	4.51 (0.95–21.0)	0.06
Q3	Comorbidity vs. No comorbidity	Moderately	1.04 (0.40–2.79)	0.94
		Quite a bit/Extremely	1.52 (0.49–4.67)	0.47
	Intravenous*^b^* vs. TKI	Moderately	0.51 (0.19–1.38)	0.18
		Quite a bit/Extremely	0.23 (0.08–0.68)	**0.008**
	Months from cancer diagnosis: (3,12] vs. ≤ 3	Moderately	0.72 (0.18–2.91)	0.64
		Quite a bit/Extremely	1.20 (0.11–13.7)	0.88
	>12 vs. ≤3	Moderately	0.83 (0.23–3.03)	0.78
		Quite a bit/Extremely	2.70 (0.29–24.8)	0.38
Q9	Previous surgery/pulmonary RT	COVID-19	1.52 (0.61–3.81)	0.37
		Both equally	0.79 (0.29–2.17)	0.64
	Intravenous*^b^* vs TKI	COVID-19	0.91 (0.36–2.30)	0.84
		Both equally	2.93 (0.85–10.1)	0.09
	Months from cancer diagnosis: (3,12] vs. ≤ 3	COVID-19	4.43 (0.61–32.7)	0.14
		Both equally	3.75 (0.92–15.2)	0.06
	12 vs. ≤3	COVID-19	6.58 (1.08–40.1)	0.04
		Both equally	1.53 (0.37–6.34)	0.56

^a^Reference levels are: not at all/a little for Q1, Q2, and Q3, Oncological disease for Q9; ^b^Intravenous: CT,CT+IO, CT+RT, IO.

TKI, tyrosine kinase inhibitors; CT, chemotherapy; IO, immunotherapy; RT, radiotherapy.

### Worries About the Impact of the COVID-19 Pandemic on Lung Cancer

#### Cancer Treatment Type and Time From Cancer Diagnosis Affect Patients’ Perception of COVID-19 Pandemic Impact on Lung Cancer

The patients’ perception of the impact of the COVID-19 pandemic on lung cancer was investigated by questions 3 and 4 (Q3-Q4). Patients were asked whether they were worried about the evolution of their cancer at the beginning of the pandemic (Q3) and at the time of our interview (Q4). Overall, 70 (45%) of patients in Q3 and 92 (59%) in Q4 reported *not at all/a little* (**Table 2**).

In univariate analysis, factors significantly associated with answer distribution were anticancer treatment type (p = 0.003), and time to cancer diagnosis (p = 0.01) (**Table 3**, note c; **Supplementary Table 4**). In particular, 18 (33%) patients receiving TKIs compared to 10 (12%) IV treatments reported being *quite a bit/extremely* worried about their cancer evolution at the beginning of the pandemic.

Similar findings were observed among patients with long-term compared to short-term cancer diagnosis both in Q3 and Q4 with 27 (28%) vs. 2 (7%) patients (Q3) and 15 (16%) vs. 2 (7%) (Q4) respectively (**Table 3**; **Supplementary Table 5**). In multivariable analysis, intravenous treatment confirmed to be significantly associated with a lower probability for patients to be *quite a bit/extremely* worried about lung cancer evolution at the beginning of the pandemic (Q3, OR = 0.23, 95% CI: (0.08–0.68), p = 0.008) (**Supplementary Figure 1**, **Table 5**)

#### Lung Cancer Patients Positively Received the Adopted Protective Measures

Questions 5 and 6 (Q5-Q6) were put to patients (N = 64) whose treatment/visit was delayed at least once during the pandemic, to investigate whether they were worried about the possibility of disease progression (Q5) or relieved about SARS-CoV-2 contagion risk reduction (Q6) with this adopted measure. Fifty-three (83%) patients reported *not at all/a little* (Q5), and 34 (53%) patients were *quite a bit/extremely* relieved by the delay (**Table 2**). The frequency of answer distribution was significantly affected by anticancer treatment type both for Q5 and Q6. A total of 29 (97%) patients receiving TKI vs. 23 (72%) IV treatments were *not at all/a little* worried (Q5, p = 0.008), while 23 (77%) patients vs. 10 (31%) patients receiving TKI and i.v. treatments respectively, reported being *quite a bit/extremely* worried (Q6, p < 0.001) (**Supplementary Table 4**). All patients were asked question 7 (Q7), to which 101 (73%) reported *not at all/a little* concerned about SARS-CoV-2 risk exposure with continuing anticancer treatment at our institute (**Table 2**).

### Impact on QoL

#### Cancer Treatment Type Affect Patients’ Perception of COVID-19 Pandemic Impact on QoL

The potential impact of the COVID-19 pandemic on quality of life (QoL) was investigated by question 8 (Q8), asking patients whether the quarantine limitations had worsened their QoL. Eighty-eight (56%) patients reported *not at all/a little* worsening of QoL (**Table 2**). However, the Q8 answer distribution was significantly affected by treatment type at univariate analysis, with 13 (24%) patients receiving TKI vs. 7 (15%) IV treatments being *moderately* to *quite a bit/extremely* worried (**Table 3**; **Supplementary Table 4**).

## Discussion

This study is the first to assess various aspects of the SARS-CoV2 pandemic impact on lung cancer patients. The aspects studied were: the fear of falling ill with COVID-19 compared to the fear of their disease, how much the COVID-19 emergency had changed patients’ lives, and whether they were more afraid of COVID-19 than lung cancer. After the end of the lockdown period, patients were interviewed. The interview explored both the time of the interview itself and the previous period of maximum contagion and restrictions. A key element revealed in the interviews was a widespread fear of the pandemic, and the persistence of this fear even after – and despite - the progressive improvement in public knowledge regarding the pandemic picture in Italy and its gradually decreasing severity over time. When the pandemic was announced, a significant percentage of patients (40%) were afraid of COVID-19. At that time, Italian news channels presented the number of deaths every day, broadcasting images of coffins with the unburied dead, intensive care units filled to over-capacity, and reporting the unavailability of swab tests, and the difficulty to get a CT scan. After the end of the most severe period and the end of the lockdown, even though the news broadcasts reported a progressive improvement of all these aspects, this did not reassure most of the patients interviewed, with fears remaining unallayed in a high percentage of patients (36%).

Analyzing the responses by subgroups, we found that those who were very afraid of COVID-19, both during and after the emergency period, were predominantly women: 55% vs. 33% and 53% vs. 26%, respectively. In discussions with patients, it emerged that women, often mothers of young children or adolescents, feel responsible for their loved ones, and the fear of infecting their parents or grandparents. In the interviews, it was often the women who spoke about the fear of becoming ill with COVID-19 and with the added risk of infecting others in the family: children, grandchildren, mothers, fathers, and spouses.

Patients with comorbidity formed the majority of those *extremely* concerned about the contagion compared to the group without comorbidities (24% vs. 9%) both in the period of maximum pandemic (24% vs. 9%) and at the end (17% vs. 5%) of April 2020. The same was found for patients who had received a “lung insult” (previous radiotherapy or surgery) compared to those who had not received such treatments (30% vs. 11%). From the interviews it emerged that about half of the patients (49%) were afraid that COVID-19 might alter the course of their tumor and also in this case, when the pandemic peak and lockdown had passed, a significant percentage of patients (36%) continued to have this fear. Patients with an older cancer diagnosis were also found to harbor such a fear. Speaking with patients, it emerged that the fear of becoming infected with the COVID-19 virus - even after the pandemic emergency period had passed - is a fear that “frail people” can’t stop carrying inside.

Many patients explained that despite the acute emergency phase being over, their fear was maintained by the failure to return to normal, the still-ongoing risk of becoming infected, and the inability to shake hands with their doctor and simply see his or her smile, both parties of course having their faces hidden beneath a mask and protective glasses.

The referral oncologists phoned each patient during the pandemic peak to discuss and explain the reasons behind postponements or modifications of therapies and scheduled visits, and explain any possible impact of such procedures on the patient’s outcome (13, 14).

These telephone calls were made before the patients received the information from the appointments secretaries regarding the logistics of the postponement of treatment/visit. Only 9% of the 64 patients said they were extremely concerned about the planned postponements/modifications. In the meantime, more than half (53%) of the patients felt reassured by these decisions because of a reduction of risk of contagion.

Moreover, after discussing the possible impact of the ongoing anticancer treatment on the risk of contagion, over 70% of the 138 treated patients did not feel more greatly exposed to the risk of infection due to cancer treatment.

These results underlined that physicians’ personalized and tailored communication enabled them to manage the possible emotional impact of such decisions. It therefore became possible to instill a state of calm for the patients, who placed their trust in the doctor’s judgment.

It should be noted that patients on oral therapy, for whom the continuity of treatment was maintained thanks to the resupply carried out at home, experienced a sense of relief from the postponement of hospital access in over 90% of cases.

A considerable proportion of those patients on intravenous therapy who needed to have treatment delayed for a few weeks to avoid the risk of viral infection reported that they were not worried about omitting treatment (72%). Indeed, many (44%) were reassured. This could underline the importance of accurately and carefully selecting the subgroup of patients to defer: patients who were well, who had had the disease under control for a long time, and who had been receiving treatment for a long time. In addition, our intervention of phone calls and sharing with our patients the choices we made, led patients to understand our position and our efforts to protect them from infection. The patients trusted us. We believe it is crucial, especially in these times of emergency - an unprecedented experience for our generation - to express to patients our difficulties and proffer our professional advice on how they may protect themselves. The value of this individualized contact with the patient is also supported by data from a study by Ghosh et al. which they investigated a population with different cultures and affected by several solid tumors and not only lung cancer (13). In this study, where the authors did not report an individualized telephone discussion to discuss and justify individualized procedures and treatment delay or modification, 68% of the patients wanted to continue treatment without postponement, despite a high percentage of patients concerned about the risk of infection (61%) (15).

In our study, patients receiving immunotherapy alone, compared with those receiving intravenous chemotherapy or immune-chemotherapy, showed no statistically significant differences in interview responses, except for the percentage of patients most frightened by COVID-19 at the time of emergency (Extremely: 26% vs. 12%). Some of the patients who were very afraid of COVID-19 stated that it was because they were scared of losing their survival advantage (“now that I’ve made it and they found the right drug!”) conferred by the immunotherapy if they got infected. Overall, quarantine worsened the quality of life in many patients (40%), especially in patients on oral therapy (47%). This was less so in those on intravenous therapy (29%). The main reason patients gave for this difference seemed to be that on intravenous treatment they had already adopted restrictive habits in their daily life, regardless of the risk of infection, so that the restrictions imposed by the pandemic did not substantially change their day-to-day living.

Other interesting issues were captured by this interview and should be considered whenever oncologists discuss with patients with cancer in this COVID-19 era, because these fears are not often made explicit.

Many patients reported that they were afraid that they might not receive treatment for COVID-19 because they had lung cancer and, in a time of emergency, a selection would be made of who would be chosen to survive COVID-19. The decision, they maintained, would surely fall in favor of healthy people.

Moreover, many patients (21%) were more worried about COVID-19 than the tumor itself. In a subgroup analysis, this appeared much more frequently in patients with a diagnosis of lung cancer made more than 12 months previously, than in patients with a recent diagnosis (<3 months). The most frequently collected reasons were manifold. The pandemic was more frightening than cancer because the virus is invisible to our senses, there is no COVID-19 standardized treatment, and it is often difficult to diagnose. Moreover, in the event of a severe infection and subsequent death from COVID-19, they would be isolated, perhaps treated, kept permanently in isolation, and then die alone without having the opportunity to embrace their loved ones. Death from cancer is often expected, and very often gives the patient more time to organize the affairs of those who remain (children, wives, husbands, family members, etc.) and above all to embrace and say their goodbyes to everyone. They would not die alone but would have their loved ones close by. Death from lung cancer, although undoubtedly tragic, would at least be experienced with less fear and less loneliness.

The results gathered from this survey can help to understand objectively the many conflicting fears harbored by lung cancer patients concerning the experience of COVID-19, the possible spread of their cancer due to the pandemic and the fear of a possible delay in treatment. It is important to share with the patients themselves, in an individualized manner, both the choices made in terms of changes regarding the pre-COVID-19 routine within the hospital and the difficulties that the doctors themselves face in an emergency crisis. Awareness improves understanding, and increases confidence in the doctor. It can also reduce the risk of contagion of patients.

## Data Availability Statement

All datasets presented in this study are included in the article/**Supplementary Material**.

## Ethics Statement

Ethical review and approval was not required for the study on human participants in accordance with the local legislation and institutional requirements. Written informed consent for participation was not required for this study in accordance with the national legislation and the institutional requirements.

## Author Contributions

CC, FM, GS, ES, IA, VS, LG, and SM designed the study, analyzed the data, and wrote the manuscript. DR, IA, CC, and GS collected and analyzed the data. CC and FM edited the manuscript. All authors had full access to all the data in the study and take responsibility for the integrity of the data and the accuracy of the data analysis. All authors contributed to the article and approved the submitted version.

## Funding

This work was partially supported by the Italian Ministry of Health with Ricerca Corrente and 5x1000 funds.

## Conflict of Interest

The authors declare that the research was conducted in the absence of any commercial or financial relationships that could be construed as a potential conflict of interest.

## Appendix 1: Interview

*During the interview, the questions asked were the following*:

**1) At the beginning of the COVID-19 emergency (February 2020), were you afraid of COVID-19?**
not at all, a little, moderately, quite a bit, extremely, not evaluable
**2) and now?**
not at all, a little, moderately, quite a bit, extremely, not evaluable
**3) At the beginning of the COVID-19 emergency (February 2020), were you afraid of the future course of your cancer due to the fear of infection from the virus?**
not at all, a little, moderately, quite a bit, extremely, not evaluable
**4) and now?**
not at all, a little, moderately, quite a bit, extremely, not evaluable
**5) Were you concerned in terms of possible disease progression when the oncologist called you to postpone your treatment/visit?**
not at all, a little, moderately, quite a bit, extremely, not evaluable
**6) Did you feel relieved in terms of reducing the risk of contagion when the oncologist called you to communicate the cancellation of your treatment/visit?**
not at all, a little, moderately, quite a bit, extremely, not evaluable
**7) Did you feel more exposed to the risk of contagion due to cancer treatment?**
not at all, a little, moderately, quite a bit, extremely, not evaluable
**8) In this precise period, how much has the limitation imposed by the quarantine worsened your quality of life compared to the pre-COVID-19 period?**
not at all, a little, moderately, quite a bit, extremely, not evaluable
**9) Are you more afraid of COVID-19 or of your cancer?**
COVID-19, cancer, both equally
