# Supplementary material for: Fears and Perception of the Impact of COVID-19 on Patients With Lung Cancer: A Mono-Institutional Survey
Source: Front Oncol. 2020 Oct 14;10:584612. doi: 10.3389/fonc.2020.584612 (PMC7591454; doi:10.3389/fonc.2020.584612)
Supplement: Supplementary file 1 [file DataSheet_1.docx]

**Supplementary Fig. 1.** Multivariable Odds Ratios for answers comparisons:

1. Moderately and Quite a bit/extremely answer levels compared to Not at all/A little for

Q1,Q2 and Q3

**(B)** COVID and Both Equally answer levels compared to Oncological Disease for Q9

| 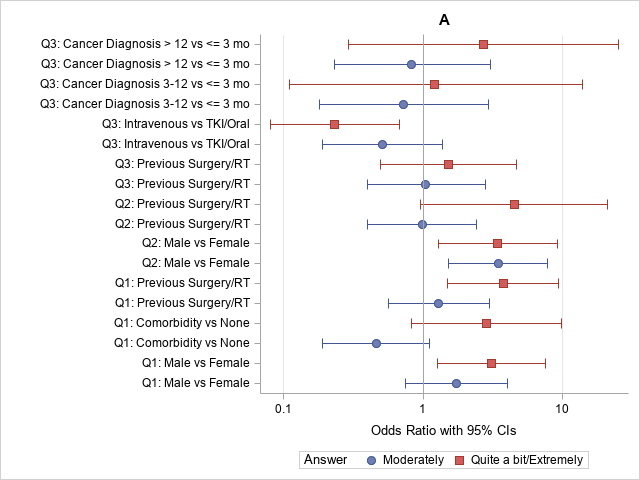 |
| --- |
| 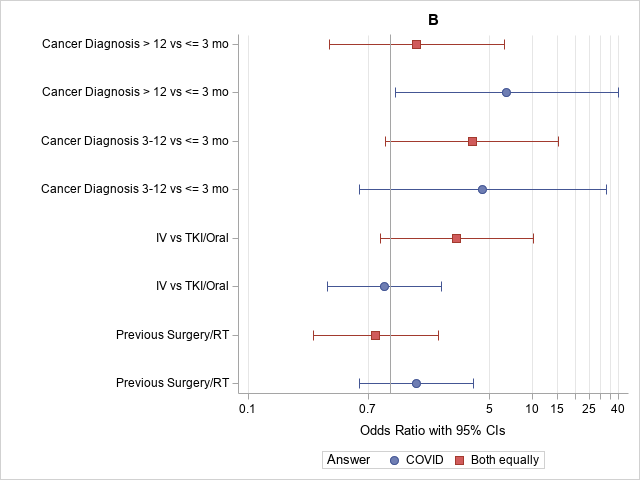 |
