# Supplementary material for: Fears and Perception of the Impact of COVID-19 on Patients With Lung Cancer: A Mono-Institutional Survey
Source: Front Oncol. 2020 Oct 14;10:584612. doi: 10.3389/fonc.2020.584612 (PMC7591454; doi:10.3389/fonc.2020.584612)
Supplement: Supplementary file 2 [file Table_1.docx]

**Supplementary Table 1.** Frequency distribution of answers to the structured interview by Comorbidities

|  |  | **Comorbidity, N (column %)** | | **p-value *^a^*** |
| --- | --- | --- | --- | --- |
| **Question** | **Level** | **No**  **N = 42** | **Yes**  **N = 114** |  |
| **Q1** | **Not at all/A little** | 20 (47.6) | 66 (57.9) |  |
|  | **Moderately** | 14 (33.3) | 19 (16.7) |  |
|  | **Quite a bit/Extremely** | 4 (9.5) | 27 (23.7) |  |
|  | ***Missing*** | 4 (9.5) | 2 (1.8) | **0.006** |
| **Q2** | **Not at all/A little** | 25 (59.5) | 69 (60.5) |  |
|  | **Moderately** | 11 (26.2) | 24 (21.1) |  |
|  | **Quite a bit/Extremely** | 2 (4.8) | 20 (17.5) |  |
|  | ***Missing*** | 4 (9.5) | 1 (0.9) | **0.01** |
| **Q3** | **Not at all/A little** | 18 (42.9) | 52 (45.6) |  |
|  | **Moderately** | 11 (26.2) | 34 (29.8) |  |
|  | **Quite a bit/Extremely** | 9 (21.4) | 23 (20.2) |  |
|  | ***Missing*** | 4 (9.5) | 5 (4.4) | 0.65 |
| **Q4** | **Not at all/A little** | 27 (64.3) | 65 (57.0) |  |
|  | **Moderately** | 7 (16.7) | 30 (26.3) |  |
|  | **Quite a bit/Extremely** | 4 (9.5) | 15 (13.2) |  |
|  | **Missing** | 4 (9.5) | 4 (3.5) | 0.27 |
| **Q5 *^b^*** | **Not at all/A little** | 15 (79.0) | 38 (84.4) |  |
|  | **Moderately** | 1 (5.3) | 3 (6.7) |  |
|  | **Quite a bit/Extremely** | 3 (15.8) | 3 (6.7) |  |
|  | ***Missing*** | 0 | 1 (2.2) | 0.55 |
| **Q6 *^b^*** | **Not at all/A little** | 6 (31.6) | 14 (31.1) |  |
|  | **Moderately** | 1 (5.3) | 7 (15.6) |  |
|  | **Quite a bit/Extremely** | 11 (57.9) | 23 (51.1) |  |
|  | ***Missing*** | 1 (5.3) | 1 (2.2) | 0.66 |
| **Q7 *^c^*** | **Not at all/A little** | 28 (71.8) | 73 (73.7) |  |
|  | **Moderately** | 3 (7.7) | 9 (9.1) |  |
|  | **Quite a bit/Extremely** | 4 (10.3) | 12 (12.1) |  |
|  | ***Missing*** | 4 (10.3) | 5 (9.1) | 0.73 |
| **Q8** | **Not at all/A little** | 19 (45.2) | 69 (60.5) |  |
|  | **Moderately** | 10 (23.8) | 18 (15.8) |  |
|  | **Quite a bit/Extremely** | 9 (21.4) | 23 (20.2) |  |
|  | **Missing** | 4 (9.5) | 4 (3.5) | 0.20 |
| **Q9** | **COVID** | 8 (19.1) | 25 (21.9) |  |
|  | **Oncological disease** | 24 (57.1) | 65 (57.0) |  |
|  | **Both equally** | 10 (16.7) | 19 (16.7) |  |
|  | ***Missing*** | 3 (7.1) | 5 (4.4) | 0.91 |

***^a^*** Fisher’s exact test (including missing values for tables with missing answers > 5%);

***^b^*** Sample Size N = 64 (delayed patients only, see text for details);

***^c^*** Sample Size N =138 (excluding subjects without therapy).
