# Supplementary material for: Fears and Perception of the Impact of COVID-19 on Patients With Lung Cancer: A Mono-Institutional Survey
Source: Front Oncol. 2020 Oct 14;10:584612. doi: 10.3389/fonc.2020.584612 (PMC7591454; doi:10.3389/fonc.2020.584612)
Supplement: Supplementary file 3 [file Table_2.docx]

**Supplementary Table 2.** Frequency distribution of answers to the structured interview by Previous Surgery/Pulmonary RT

|  |  | **Previous Surgery/Pulmonary RT, N (column %)** | | **p-value *^a^*** |
| --- | --- | --- | --- | --- |
| **Question** | **Level** | **No**  **N = 83** | **Yes**  **N = 73** |  |
| **Q1** | **Not at all/A little** | 52 (62.7) | 34 (46.6) |  |
|  | **Moderately** | 17 (20.5) | 16 (21.9) |  |
|  | **Quite a bit/Extremely** | 9 (10.8) | 22 (30.1) |  |
|  | ***Missing*** | 5 (6.0) | 1 (1.4) | **0.01** |
| **Q2** | **Not at all/A little** | 54 (68.4) | 40 (55.6) |  |
|  | **Moderately** | 16 (20.3) | 19 (26.4) |  |
|  | **Quite a bit/Extremely** | 9 (11.4) | 13 (18.1) |  |
|  | **Missing** | 4 (4.8) | 1 (1.4) | 0.26 |
| **Q3** | **Not at all/A little** | 38 (45.8) | 32 (43.8) |  |
|  | **Moderately** | 24 (28.9) | 21 (28.8) |  |
|  | **Quite a bit/Extremely** | 13 (15.7) | 19 (26.0) |  |
|  | ***Missing*** | 8 (9.6) | 1 (1.4) | 0.08 |
| **Q4** | **Not at all/A little** | 45 (54.2) | 47 (64.4) |  |
|  | **Moderately** | 20 (24.1) | 17 (23.3) |  |
|  | **Quite a bit/Extremely** | 11 (13.3) | 8 (11.0) |  |
|  | **Missing** | 7 (8.4) | 1 (1.4) | 0.20 |
| **Q5 *^b^*** | **Not at all/A little** | 27 (79.4) | 26 (86.7) |  |
|  | **Moderately** | 2 (5.9) | 2 (6.7) |  |
|  | **Quite a bit/Extremely** | 5 (14.7) | 1 (3.3) |  |
|  | ***Missing*** | 0 | 1 (3.3) | 0.34 |
| **Q6 *^b^*** | **Not at all/A little** | 12 (35.3) | 8 (26.7) |  |
|  | **Moderately** | 4 (11.8) | 4 (13.3) |  |
|  | **Quite a bit/Extremely** | 18 (52.9) | 16 (53.3) |  |
|  | ***Missing*** | 0 | 2 (6.7) | 0.53 |
| **Q7 *^c^*** | **Not at all/A little** | 61 (82.4) | 40 (62.5) |  |
|  | **Moderately** | 3 (4.1) | 9 (14.1) |  |
|  | **Quite a bit/Extremely** | 6 (8.1) | 10 (15.6) |  |
|  | ***Missing*** | 4 (5.4) | 5 (7.8) | 0.05 |
| **Q8** | **Not at all/A little** | 52 (62.6) | 36 (49.3) |  |
|  | **Moderately** | 13 (15.7) | 15 (20.6) |  |
|  | **Quite a bit/Extremely** | 12 (14.5) | 20 (27.4) |  |
|  | ***Missing*** | 6 (7.2) | 2 (2.7) | 0.10 |
| **Q9** | **COVID** | 11 (13.3) | 22 (30.1) |  |
|  | **Oncological disease** | 48 (57.8) | 41 (56.2) |  |
|  | **Both equally** | 16 (19.3) | 10 (13.7) |  |
|  | ***Missing*** | 8 (9.6) | 0 | **0.003** |

***^a^*** Fisher’s exact test (including missing values for tables with missing answers > 5%);

***^b^*** Sample Size N = 64 (delayed patients only, see text for details);

***^c^*** Sample Size N =138 (excluding subjects without therapy); RT= Radiotherapy.
