# Supplementary material for: Fears and Perception of the Impact of COVID-19 on Patients With Lung Cancer: A Mono-Institutional Survey
Source: Front Oncol. 2020 Oct 14;10:584612. doi: 10.3389/fonc.2020.584612 (PMC7591454; doi:10.3389/fonc.2020.584612)
Supplement: Supplementary file 4 [file Table_3.docx]

**Supplementary Table 3.** Frequency distribution of answers to the structured interview by Gender

|  |  | **Gender, N (column %)** | | **p-value *^a^*** |
| --- | --- | --- | --- | --- |
| **Question** | **Level** | **Female**  **N = 69** | **Male**  **N = 87** |  |
| **Q1** | **Not at all/A little** | 30 (45.5) | 56 (66.7) |  |
|  | **Moderately** | 17 (25.8) | 16 (19.1) |  |
|  | **Quite a bit/Extremely** | 19 (28.8) | 12 (14.3) |  |
|  | ***Missing*** | 3 (4.4) | 3 (4.4) | **0.02** |
| **Q2** | **Not at all/A little** | 31 (47.0) | 63 (74.1) |  |
|  | **Moderately** | 22 (33.3) | 13 (15.3) |  |
|  | **Quite a bit/Extremely** | 13 (19.7) | 9 (10.6) |  |
|  | ***Missing*** | 3 (4.4) | 2 (2.3) | **0.003** |
| **Q3** | **Not at all/A little** | 25 (36.2) | 45 (51.7) |  |
|  | **Moderately** | 20 (29.0) | 25 (28.7) |  |
|  | **Quite a bit/Extremely** | 18 (26.1) | 14 (16.1) |  |
|  | ***Missing*** | 6 (8.7) | 3 (3.5) | 0.12 |
| **Q4** | **Not at all/A little** | 35 (50.7) | 57 (65.5) |  |
|  | **Moderately** | 18 (26.1) | 19 (21.8) |  |
|  | **Quite a bit/Extremely** | 10 (14.5) | 9 (10.3) |  |
|  | **Missing** | 6 (8.7) | 2 (2.3) | 0.14 |
| **Q5 *^b^*** | **Not at all/A little** | 25 (80.7) | 28 (84.9) |  |
|  | **Moderately** | 3 (9.7) | 1 (3.0) |  |
|  | **Quite a bit/Extremely** | 3 (9.7) | 3 (9.1) |  |
|  | ***Missing*** | 0 | 1 (3.0) | 0.60 |
| **Q6 *^b^*** | **Not at all/A little** | 6 (12.4) | 14 (42.2) |  |
|  | **Moderately** | 6 (12.4) | 2 (6.1) |  |
|  | **Quite a bit/Extremely** | 19 (61.3) | 15 (45.5) |  |
|  | ***Missing*** | 0 | 2 (6.1) | 0.05 |
| **Q7 *^c^*** | **Not at all/A little** | 41 (65.1) | 60 (80.0) |  |
|  | **Moderately** | 9 (14.3) | 3 (4.0) |  |
|  | **Quite a bit/Extremely** | 7 (11.1) | 9 (12.0) |  |
|  | ***Missing*** | 6 (9.5) | 3 (4.0) | 0.08 |
| **Q8** | **Not at all/A little** | 36 (52.2) | 52 (59.8) |  |
|  | **Moderately** | 15 (21.7) | 13 (14.9) |  |
|  | **Quite a bit/Extremely** | 14 (20.3) | 18 (20.7) |  |
|  | ***Missing*** | 4 (5.8) | 4 (4.6) | 0.67 |
| **Q9** | **COVID** | 14 (20.3) | 19 (21.8) |  |
|  | **Oncological disease** | 44 (63.8) | 45 (51.7) |  |
|  | **Both equally** | 10 (14.5) | 16 (18.4) |  |
|  | ***Missing*** | 4 (1.5) | 7 (8.1) | 0.21 |

***^a^*** Fisher’s exact test (including missing values for tables with missing answers > 5%);

***^b^*** Sample Size N = 64 (delayed patients only, see text for details);

***^c^*** Sample Size N =138 (excluding subjects without therapy).
