# Supplementary material for: Fears and Perception of the Impact of COVID-19 on Patients With Lung Cancer: A Mono-Institutional Survey
Source: Front Oncol. 2020 Oct 14;10:584612. doi: 10.3389/fonc.2020.584612 (PMC7591454; doi:10.3389/fonc.2020.584612)
Supplement: Supplementary file 5 [file Table_4.docx]

**Supplementary Table 4.** Frequency distribution of answers to the structured interview TKI vs IV therapies comparison

|  |  | **Therapy *^a^*,**  **N (column %)** | |  |
| --- | --- | --- | --- | --- |
| **Question** | **Level** | **Intravenous *^b^***  **N = 83** | **TKI**  **N = 55** | **p-value *^c^*** |
| **Q1** | **Not at all/A little** | 52 (62.7) | 24 (43.6) |  |
|  | **Moderately** | 14 (16.9) | 14 (25.5) |  |
|  | **Quite a bit/Extremely** | 15 (18.1) | 14 (25.5) |  |
|  | ***Missing*** | 2 (2.4) | 3 (5.5) | 0.15 |
| **Q2** | **Not at all/A little** | 49 (59.0) | 34 (61.8) |  |
|  | **Moderately** | 17 (20.5) | 13 (23.6) |  |
|  | **Quite a bit/Extremely** | 16 (19.3) | 5 (9.1) |  |
|  | ***Missing*** | 1 (1.2) | 3 (5.5) | 0.21 |
| **Q3** | **Not at all/A little** | 48 (57.8) | 17 (30.9) |  |
|  | **Moderately** | 22 (26.5) | 15 (27.3) |  |
|  | **Quite a bit/Extremely** | 10 (12.1) | 18 (32.7) |  |
|  | ***Missing*** | 3 (2.1) | 5 (9.1) | **0.003** |
| **Q4** | **Not at all/A little** | 55 (60.4) | 28 (50.9) |  |
|  | **Moderately** | 19 (27.1) | 12 (21.8) |  |
|  | **Quite a bit/Extremely** | 7 (10.4) | 10 (18.2) |  |
|  | ***Missing*** | 2 (2.4) | 5 (9.1) | 0.08 |
| **Q5 *^d^*** | **Not at all/A little** | 23 (71.9) | 29 (96.7) |  |
|  | **Moderately** | 4 (12.5) | 0 |  |
|  | **Quite a bit/Extremely** | 5 (15.6) | 0 |  |
|  | ***Missing*** | 0 | 1 (3.3) | **0.008** |
| **Q6 *^d^*** | **Not at all/A little** | 17 (53.1) | 2 (6.7) |  |
|  | **Moderately** | 4 (12.5) | 4 (13.3) |  |
|  | **Quite a bit/Extremely** | 10 (31.3) | 23 (76.7) |  |
|  | ***Missing*** | 1 (3.1) | 1 (3.3) | **< 0.001** |
| **Q7** | **Not at all/A little** | 66 (79.5) | 35 (63.6) |  |
|  | **Moderately** | 9 (10.8) | 3 (5.5) |  |
|  | **Quite a bit/Extremely** | 7 (8.4) | 9 (16.4) |  |
|  | ***Missing*** | 1 (1.2) | 8 (14.6) | **0.004 *^e^*** |
| **Q8** | **Not at all/A little** | 34 (70.8) | 25 (45.5) |  |
|  | **Moderately** | 7 (14.6) | 13 (23.6) |  |
|  | **Quite a bit/Extremely** | 7 (14.6) | 13 (23.6) |  |
|  | ***Missing*** | 0 | 4 (7.3) | **0.04** |
| **Q9** | **COVID** | 5 (10.4) | 14 (25.5) |  |
|  | **Oncological disease** | 30 (62.5) | 35 (63.6) |  |
|  | **Both equally** | 11 (27.1) | 4 (7.3) |  |
|  | ***Missing*** | 2 (4.2) | 2 (3.6) | **0.02** |

***^a^*** Sample Size N =138 (excluding subjects without therapy); ***^b^*** Intravenous: CT,CT+IO,CT+RT,IO

***^c^*** Fisher’s exact test (including missing values for tables with missing answers > 5%); ***^d^*** Sample Size N = 62 (delayed treatment patients only, see text for details); ***^e^*** p = 0.18 exlcuding missing answers

TKI= Tyrosine Kinase Inhibitor; CT = Chemotherapy; IO = Immunotherapy; RT = Radiotherapy;

IV= Intravenous.
