# Supplementary material for: Fears and Perception of the Impact of COVID-19 on Patients With Lung Cancer: A Mono-Institutional Survey
Source: Front Oncol. 2020 Oct 14;10:584612. doi: 10.3389/fonc.2020.584612 (PMC7591454; doi:10.3389/fonc.2020.584612)
Supplement: Supplementary file 6 [file Table_5.docx]

**Supplementary Table 5.** Frequency distribution of answers to the structured interview by time from cancer diagnosis

|  |  | **Months from cancer diagnosis**  **N (column %)** | | | **p-value *^a^*** |
| --- | --- | --- | --- | --- | --- |
| **Question** | **Level** | **≤ 3**  **N = 28** | **(3,12]**  **N = 32** | **> 12**  **N = 96** |  |
| **Q1** | **Not at all/A little** | 17 (60.7) | 22 (68.8) | 47 (49.0) |  |
|  | **Moderately** | 8 (28.6) | 3 (9.4) | 22 (22.9) |  |
|  | **Quite a bit/Extremely** | 1 (3.6) | 6 (18.8) | 24 (25.0) |  |
|  | ***Missing*** | 2 (7.1) | 1 (3.1) | 3 (3.1) | 0.05 |
| **Q2** | **Not at all/A little** | 17 (60.7) | 23 (71.9) | 54 (56.3) |  |
|  | **Moderately** | 6 (21.4) | 5 (15.6) | 24 (25.0) |  |
|  | **Quite a bit/Extremely** | 3 (10.7) | 3 (9.4) | 16 (16.7) |  |
|  | **Missing** | 2 (7.1) | 1 (3.1) | 2 (2.1) | 0.55 |
| **Q3** | **Not at all/A little** | 11 (39.3) | 19 (59.4) | 40 (41.7) |  |
|  | **Moderately** | 10 (35.7) | 9 (28.1) | 26 (27.1) |  |
|  | **Quite a bit/Extremely** | 2 (7.1) | 3 (9.4) | 27 (28.1) |  |
|  | ***Missing*** | 5 (17.9) | 1 (3.1) | 3 (3.1) | **0.01** |
| **Q4** | **Not at all/A little** | 13 (46.4) | 24 (75.0) | 55 (57.3) |  |
|  | **Moderately** | 8 (28.6) | 5 (15.6) | 24 (25.0) |  |
|  | **Quite a bit/Extremely** | 2 (7.1) | 2 (6.3) | 15 (15.6) |  |
|  | ***Missing*** | 5 (17.9) | 1 (3.1) | 2 (2.1) | **0.03** |
| **Q5 *^b^*** | **Not at all/A little** | 0 | 7 (77.8) | 46 (86.8) |  |
|  | **Moderately** | 1 (50.0) | 0 | 3 (5.7) |  |
|  | **Quite a bit/Extremely** | 0 | 2 (22.2) | 3 (5.7) |  |
|  | ***Missing*** | 1 (50.0) | 0 | 1 (1.9) | 0.32 ***^d^*** |
| **Q6 *^b^*** | **Not at all/A little** | 2 (100) | 4 (44.4) | 14 (26.4) |  |
|  | **Moderately** | 0 | 0 | 8 (15.1) |  |
|  | **Quite a bit/Extremely** | 0 | 4 (44.4) | 30 (56.6) |  |
|  | ***Missing*** | 0 | 1 (11.1) | 1 (1.9) | 0.14 ***^d^*** |
| **Q7 *^c^*** | **Not at all/A little** | 12 (63.2) | 26 (86.7) | 63 (70.8) |  |
|  | **Moderately** | 1 (5.3) | 1 (3.1) | 10 (11.2) |  |
|  | **Quite a bit/Extremely** | 2 (10.5) | 3 (10.0) | 10 (11.2) |  |
|  | ***Missing*** | 4 (21.1) | 0 | 5 (5.6) | 0.13 |
| **Q8** | **Not at all/A little** | 18 (64.3) | 21 (65.6) | 49 (51.0) |  |
|  | **Moderately** | 6 (21.4) | 4 (12.5) | 18 (18.8) |  |
|  | **Quite a bit/Extremely** | 1 (3.6) | 6 (18.8) | 25 (26.0) |  |
|  | ***Missing*** | 3 (10.7) | 1 (3.1) | 4 (4.2) | 0.10 |
| **Q9** | **COVID** | 2 (7.1) | 4 (12.5) | 27 (28.1) |  |
|  | **Oncological disease** | 20 (71.4) | 14 (43.8) | 55 (57.3) |  |
|  | **Both equally** | 5 (17.9) | 10 (31.3) | 11 (11.5) |  |
|  | ***Missing*** | 1 (3.6) | 4 (12.5) | 3 (3.1) | **0.006** |

***^a^*** Fisher’s exact test (including missing values for tables with missing answers > 5%); ***^b^*** Sample Size N = 64 (delayed patients only, see text for details); ***^c^*** Sample Size N =138 (excluding subjects without therapy);

***^d^*** Significance test for (3,12) months vs > 12 months comparison only
